# Phytohormone profiles in non-transformed and *AtCKX* transgenic centaury (*Centaurium erythraea* Rafn) shoots and roots in response to salinity stress in vitro

**DOI:** 10.1038/s41598-021-00866-7

**Published:** 2021-11-02

**Authors:** Milana Trifunović-Momčilov, Václav Motyka, Petre I. Dobrev, Marija Marković, Snežana Milošević, Slađana Jevremović, Ivana Č. Dragićević, Angelina Subotić

**Affiliations:** 1 Department for Plant Physiology, Institute for Biological Research “Siniša Stanković” – National Institute of Republic of Serbia, University of Belgrade, Bulevar despota Stefana 142, Belgrade, 11060 Serbia; 2 Institute of Experimental Botany of the Czech Academy of Sciences, Rozvojová 263, 16502 Prague 6, Czech Republic; 3 Faculty of Biology, University of Belgrade, Studentski trg 16, Belgrade, 11000 Serbia

**Keywords:** Auxin, Cytokinin, Jasmonic acid, Plant physiology, Salt

## Abstract

Plant hormones regulate numerous developmental and physiological processes. Abiotic stresses considerably affect production and distribution of phytohormones as the stress signal triggers. The homeostasis of plant hormones is controlled by their de novo synthesis and catabolism. The aim of this work was to analyse the contents of total and individual groups of endogenous cytokinins (CKs) as well as indole-3-acetic acid (IAA) in *AtCKX* overexpressing centaury plants grown in vitro on graded NaCl concentrations (0, 50, 100, 150, 200 mM). The levels of endogenous stress hormones including abscisic acid (ABA), salicylic acid (SA) and jasmonic acid (JA) were also detected. The elevated contents of total CKs were found in all analysed centaury shoots. Furthermore, increased amounts of all five CK groups, as well as enhanced total CKs were revealed on graded NaCl concentrations in non-transformed and *AtCKX* roots. All analysed *AtCKX* centaury lines exhibited decreased amounts of endogenous IAA in shoots and roots. Consequently, the IAA/bioactive CK forms ratios showed a significant variation in the shoots and roots of all *AtCKX* lines. In shoots and roots of both non-transformed and *AtCKX* transgenic centaury plants, salinity was associated with an increase of ABA and JA and a decrease of SA content.

## Introduction

Plants are sedentary organisms that face numerous survival challenges such as abiotic (salinity, drought, heat, cold etc.) and biotic stresses (infection by pathogenic bacteria, viruses, fungi etc.). Among numerous environmental hindrances, abiotic stress is known as the significant factor reducing the average yield. Salinization represents one of the major sources of all abiotic stresses damaging at least 20% of the crop production all around the world^1^. Exposure of plants to salt stress results in changes in almost all physiological and biochemical processes leading to a disturbance of normal growth and development. During evolution, plants have developed different mechanisms for successful adaptation on unfavourable environmental conditions. The response of plants to stress conditions depends on multiple factors, among which phytohormones play a crucial role. Thanks to signal transduction and pathway crosstalks, phytohormones defend plants by promoting specific protective mechanisms against abiotic and biotic stresses^2^.

Plant growth and development are regulated by phytohormones such as cytokinins (CKs), auxins, abscisic acid (ABA), gibberellins, ethylene, salicylic acid (SA), brassinosteroids and jasmonates which control numerous physiological and biochemical procceses^3^. Abiotic stresses often lead to altered production and distribution of endogenous phytohormones making them stress signal triggers^4^.

Various plant growth and developmental processes are regulated by CKs. This group of phytohormones primarily regulates cell division, apical dominance, vascular differentiation, leaf senescence, chloroplast biogenesis, shoot differentiation and anthocyanin production^5^. It has also been reported that CKs are involved in enhancing tolerance against high salinity and temperature stress in plants^6^. Generally, it is accepted that CKs are predominantly produced in the root tips and developing seeds. After biosynthesis CKs are further translocated from the roots via xylem to the shoots where they regulate plant development and growth processes. The increased activity of the enzyme cytokinin oxidase/dehydrogenase (CKX, EC 1.5.99.12) is directly related to decreased amount of CK. This catabolic enzyme represent a key factor in controlling of CK levels in plant tissues. The free bases, iP and *trans*-zeatin and their respective ribosides are the preferred CKX substrates^7^, but *cis-*zeatin and its riboside have also recently been found to be efficiently degraded by some CKX isoforms^8^. In different plant species, small gene families encode CKX isoforms with varying number of members. Seven *A. thaliana CKX* genes (*AtCKX1-7*) were described almost twenty years ago^9^. It is also known that individual members of the *AtCKX* gene family are expressed differentially and have different subcellular localizations^10^. The *AtCKX1* expression is specific for shoot apex of lateral shoot meristems while the *AtCKX2* promoter is expressed in the shoot apex and not in Arabidopsis roots. On the other hand, AtCKX1 is a mitochondrial protein with vacuole as final destination while AtCKX2 represent the peptide for targeting to the endoplasmic reticulum and subsequent transport to extracellular region.

When the main plant organs, roots, are exposed to salt, they are clearly affected by salinity stress. Under salt stress conditions, a reduction of CK biosynthesis in the roots directly affects CK amounts in the shoots^11,12^. Previously it was shown that CKs increase plant tolerance to salinity stress^3,6^. These literature data, however, considered exogenous application of CKs. On the other hand, only few literature data exist describing how plants with genetically modified levels of endogenous CKs respond to salt stress^12–15^. Considering that some literature data describe both positive and negative influences of CKs on stress tolerance, all of these pieces of information could be crucial for better knowledge of CK involvement in the plant stress tolerance responses.

Auxins represent the plant hormones which promote plant growth and development. Indole-3-acetic acid (IAA) as the major auxin representative in plants mainly regulates vascular tissue development, cell elongation and apical dominance^16^. Literature data indicating involvement of auxin in response to salinity stress are somewhat limited. The root architecture of *Arabidopsis thaliana* was substantially remodelled by auxin accumulation and its redistribution under salt stress^17,18^. Some reports are also describing a significant reduction of IAA level in salt-stressed rice and tomato plants^19^. Besides, salinity was found to decrease contents of IAA and the non-indole auxin, phenylacetic acid, in both cultivated glycophyte tomato *Solanum lycopersicum* as well as in its halophyte wild relative *S. chilense*^20^.

ABA, commonly known as the stress hormone, is probably the most studied phytohormone because of response and distinct role in plant adaptation to multiple abiotic stresses^21^. It is known that under drought or salinity stress conditions, the level of endogenous ABA was significantly increased^22–24^. In different plant developmental processes such as stomatal opening in bean leaves and seed germination of Arabidopsis, CKs act as ABA antagonists^25,26^. As both these hormones individually play essential roles in stress tolerance, mainly during drought and salinity stress, a crosstalk among CKs and ABA seems to be very important for improving abiotic stress tolerance^27^. During last ten years, it was shown that CK receptors known as Arabidopsis Histidine Kinases (AHKs) represent crucial regulators during salt, drought and cold stress conditions^28,29^. In contrast to AHK1 known as a positive regulator of ABA signaling, AHK2 and AHK3 were assigned as negative regulators^11,29^. Moreover, the expression of maize *ckx1* gene was strongly affected by CKs and ABA under the conditions of abiotic stress^30^. Functional analysis of CK-deficient Arabidopsis plants showed that overexpression of *ipt* and *ckx* genes was repressed by stress and ABA treatments, leading to decreased concentrations of biologically active CKs^12,13^.

SA, endogenous plant growth regulator with phenolic nature, is involved in the regulation of physiological processes such as growth, photosynthesis, and nitrogen metabolism^31^. It also plays an important role in protection against biotic and abiotic stresses including salinity^32^. The role of SA in plant defense from salt stress conditions is species-specific and has been well documented^33–35^. For example, the endogenous SA level was decreased under salt stress in rice seedlings^36^ and salt-stressed *Iris hexagona* plants^37^. Salinity also strongly decreased SA concentration in glycophyte tomato *S. lycopersicum* but on the other hand, SA content increased in response to salt in the wild halophyte *S. chilense*^20^.

Jasmonic acid (JA) is an important cellular regulator involved in diverse developmental processes such as seed germination, root growth, fertility, fruit ripening, and senescence^37^. It is known that JA and its derivatives also strongly respond to salinity stress conditions^38^. It has been reported that JA levels in various tomato cultivars changed in response to the salt stress^39^. When exposed to salinisation, the salt-tolerant tomato cultivar considerably increased its JA level whereas the salt-sensitive cultivar decreased it. Concentration of jasmonates was more than two times higher in the halophyte *S. chilense* than in the glycophyte *S. lycopersicum*, and salinity increased its concentration by more than 100% in both species compared to control plants^20^.

Previous work described salinity stress response of non-transformed and *AtCKX* transgenic centaury shoots and roots grown in vitro^15^. It was shown that centaury roots showed higher salinity tolerance compared to shoots. Morphogenic potential of *AtCKX1* transgenic line was similar to non-transformed line under increased NaCl concentrations in vitro. On the other hand, there were significant difference in morphogenic response of two investigated *AtCKX2* transgenic lines, specially in root culture. Accordingly, in this followed work, we compared the profiles of endogenous of phytohormones in non-transformed and transgenic centaury *AtCKX* lines grown in vitro under salinity stress conditions. The contents of CKs, auxins, ABA, JA and SA were determined in shoots and roots cultured in vitro for eight weeks on increased NaCl concentrations. This work represents the first report on altered phytohormone levels in centaury, the significant medicinal plant species, during salt stress. The obtained results might be a useful tool for better understanding of salinity tolerance of non-transformed and CK-deficient transgenic centaury plants grown in vitro.

## Results

### Endogenous cytokinins

**Figure 1:**
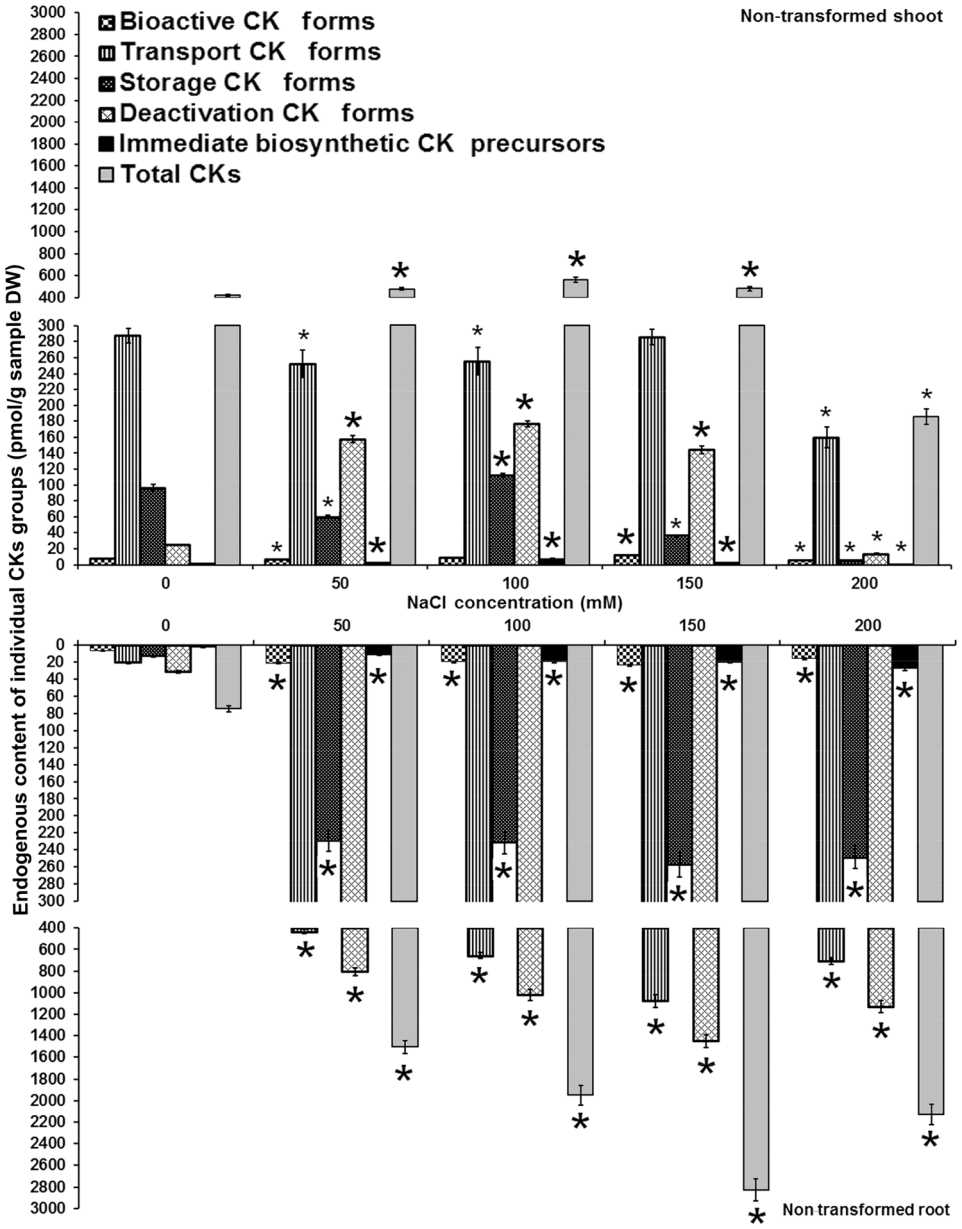
Endogenous cytokinin content in shoots and roots of 8-week-old non-transformed *Centaurium erythraea* line. According to physiological function and conjugation status cytokinins were divided into five groups including bioactive forms (DHZ, iP, *tZ*, *c*Z), transport forms (DHZ9R, iP9R, *t*Z9R, *c*Z9R), storage forms (DHZ9ROG, DHZOG, *t*Z9ROG, *t*ZOG, *c*Z9ROG, *c*ZOG), deactivation forms (DHZ7G, DHZ9G, iP7G, iP9G, *t*Z7G, *t*Z9G, *c*Z7G, *c*Z9G), immediate biosynthetic precursors (*t*ZRMP, DHZRMP, *c*ZRMP, iPRMP) and total CKs content. Data represent mean ± standard error. Means marked with asterisks are significantly different from corresponding control values (LSD test, *p* ≤ 0.05). The bigger asterisks represent the values significantly higher than control ones while smaller asterisks represent values significantly lower than control ones.

**Figure 2:**
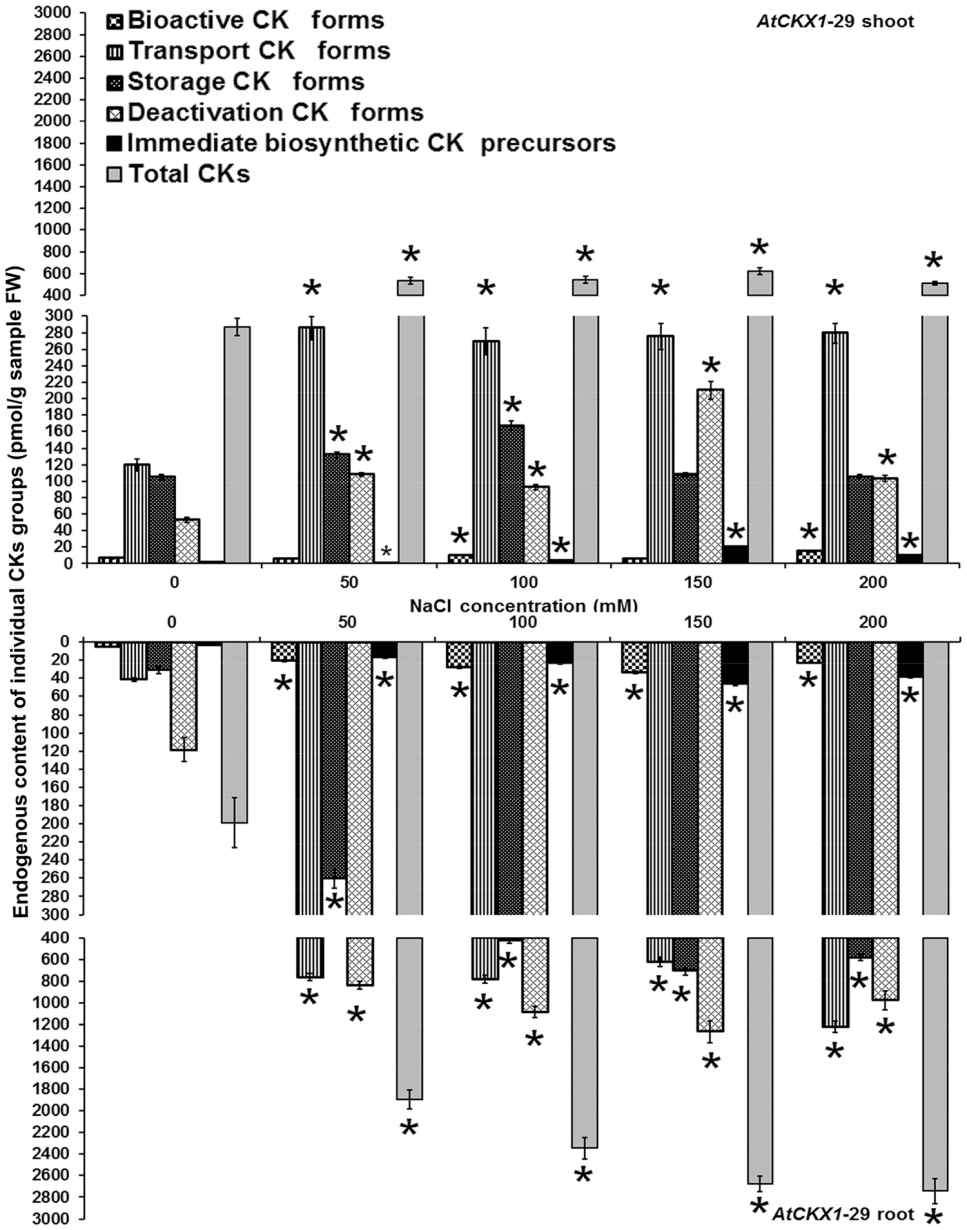
Endogenous cytokinin content in shoots and roots of 8-week-old *AtCKX1*-29 transgenic *Centaurium erythraea* line. According to physiological function and conjugation status cytokinins were divided into five groups including bioactive forms (DHZ, iP, *tZ*, *c*Z), transport forms (DHZ9R, iP9R, *t*Z9R, *c*Z9R), storage forms (DHZ9ROG, DHZOG, *t*Z9ROG, *t*ZOG, *c*Z9ROG, *c*ZOG), deactivation forms (DHZ7G, DHZ9G, iP7G, iP9G, *t*Z7G, *t*Z9G, *c*Z7G, *c*Z9G), immediate biosynthetic precursors (*t*ZRMP, DHZRMP, *c*ZRMP, iPRMP) and total CKs content (boja kolone). Data represent mean ± standard error. Means marked with asterisks are significantly different from corresponding control values (LSD test, *p* ≤ 0.05). The bigger asterisks represent the values significantly higher than control ones while smaller asterisks represent values significantly lower than control ones.

**Figure 3:**
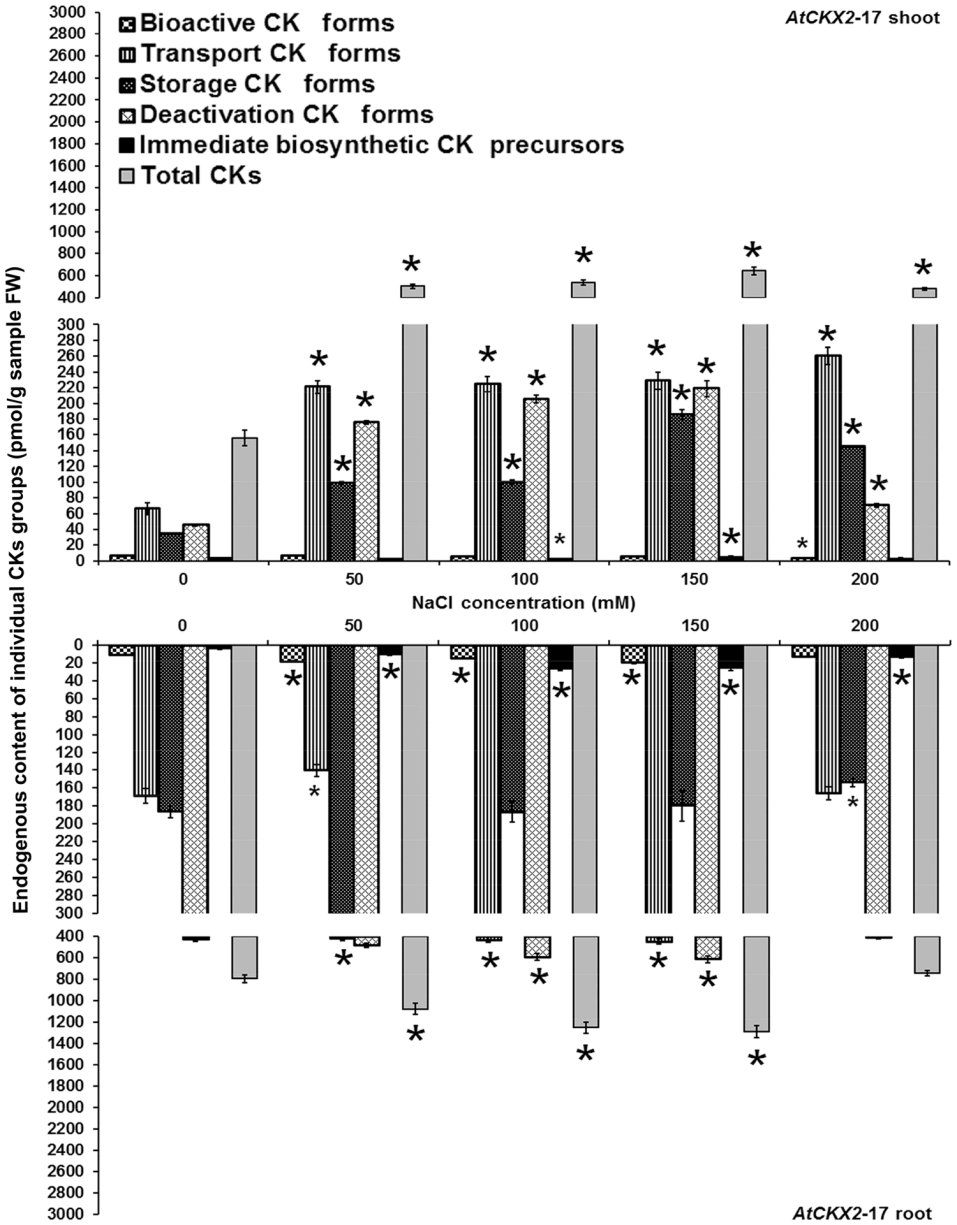
Endogenous cytokinin content in shoots and roots of 8-week-old *AtCKX2*-17 transgenic *Centaurium erythraea* line. According to physiological function and conjugation status cytokinins were divided into five groups including bioactive forms (DHZ, iP, *tZ*, *c*Z), transport forms (DHZ9R, iP9R, *t*Z9R, *c*Z9R), storage forms (DHZ9ROG, DHZOG, *t*Z9ROG, *t*ZOG, *c*Z9ROG, *c*ZOG), deactivation forms (DHZ7G, DHZ9G, iP7G, iP9G, *t*Z7G, *t*Z9G, *c*Z7G, *c*Z9G), immediate biosynthetic precursors (*t*ZRMP, DHZRMP, *c*ZRMP, iPRMP) and total CKs content (boja kolone). Data represent mean ± standard error. Means marked with asterisks are significantly different from corresponding control values (LSD test, *p* ≤ 0.05). The bigger asterisks represent the values significantly higher than control ones while smaller asterisks represent values significantly lower than control ones.

**Figure 4:**
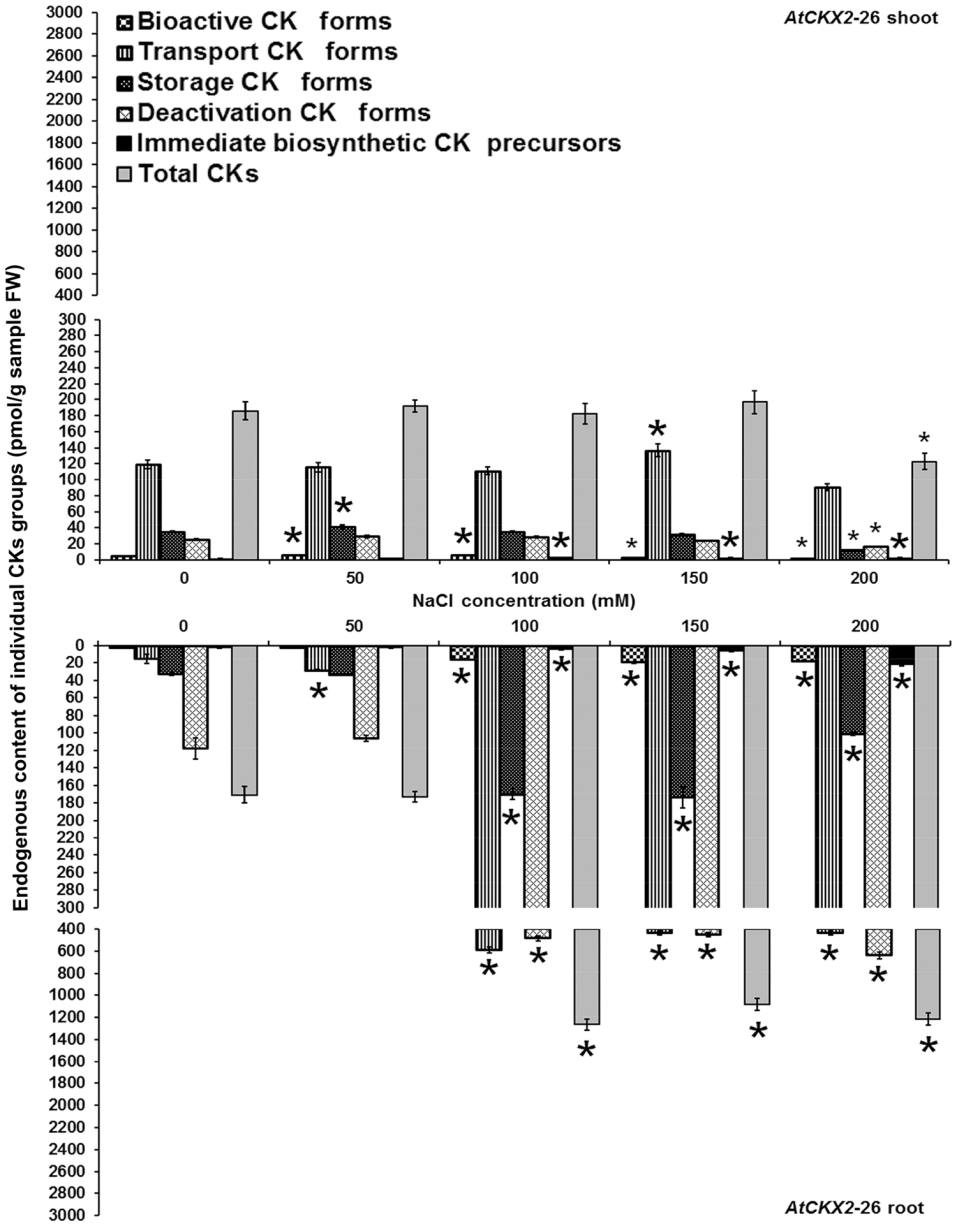
Endogenous cytokinin content in shoots and roots of 8-week-old *AtCKX2*-26 transgenic *Centaurium erythraea* line. According to physiological function and conjugation status cytokinins were divided into five groups including bioactive forms (DHZ, iP, *tZ*, *c*Z), transport forms (DHZ9R, iP9R, *t*Z9R, *c*Z9R), storage forms (DHZ9ROG, DHZOG, *t*Z9ROG, *t*ZOG, *c*Z9ROG, *c*ZOG), deactivation forms (DHZ7G, DHZ9G, iP7G, iP9G, *t*Z7G, *t*Z9G, *c*Z7G, *c*Z9G), immediate biosynthetic precursors (*t*ZRMP, DHZRMP, *c*ZRMP, iPRMP) and total CKs content (boja kolone). Data represent mean ± standard error. Means marked with asterisks are significantly different from corresponding control values (LSD test, *p* ≤ 0.05). The bigger asterisks represent the values significantly higher than control ones while smaller asterisks represent values significantly lower than control ones.

Endogenous CKs were detected and quantified in shoots and roots of non-transformed (Fig. 1), one transgenic *AtCKX1* (line 29, Fig. 2) and two *AtCKX2* (lines 17 and 26) centaury lines (Figs. 3, 4) grown on media with graded NaCl concentrations. According to their physiological function and conjugation status, endogenous CKs were divided into five groups (as proposed by^40^) including bioactive forms (CK nucleobases), transport forms (CK ribosides), storage forms (CK *O*-glucosides), deactivation forms (CK *N*-glucosides) and immediate biosynthetic precursors (CK phosphates). The abbreviations for CKs presented in this work were adopted and modified according to^41^. The levels of individual endogenous CK groups were determined and are shown on Figs. 1, 2, 3 and 4.

Analysis of endogenous CKs in non-transformed shoots showed that the levels of bioactive CK forms were significantly increased only on one NaCl concentration (150 mM) while decreased on two NaCl doses (50 and 200 mM). In non-transformed roots, the contents of bioactive CK forms were significantly enhanced on all NaCl concentrations (Fig. 1). Transport CK forms were reduced in non-transformed shoots on media supplemented with NaCl (except for 150 mM) but elevated on all media in non-transformed roots. Concentrations of storage CK forms were decreased in non-transformed shoots on all salt-treated media except for 100 mM NaCl while in roots they were considerably enhanced. Compared to control NaCl-free medium, the deactivation CK forms and CK phosphates (immediate biosynthetic CK precursors) were increased in non-transformed shoots grown on 50, 100 and 150 mM NaCl whereas on media supplemented with 200 mM NaCl their levels were decreased. In roots, deactivation CK forms and immediate biosynthetic CK precursors were increased on all applied NaCl concentrations. Increase in the total CK contents was recorded in non-transformed shoots on 50, 100 and 150 mM NaCl. On the other hand, elevated total CK levels were detected on all NaCl concentrations.

Transgenic *AtCKX1*-29 shoots showed increased levels of bioactive CKs on media containing 100 and 200 mM NaCl while their enhanced contents were found on all NaCl concentrations in roots (Fig. 2). Transport and deactivation CK forms as well as CK phosphates were increased in both *AtCKX1*-29 shoots and roots on all NaCl concentrations. The levels of storage CKs were elevated in *AtCKX1*-29 shoots only on lower, i.e. 50 and 100 mM NaCl, concentrations whereas increased storage CKs contents were recorded for all NaCl-treated variants in roots. On all NaCl concentrations, the *AtCKX1*-29 shoots and roots showed increased levels of total CKs in comparison to the control NaCl-free medium.

In shoots of transgenic *AtCKX2*-17 line, the levels of bioactive CKs were not much affected by NaCl treatments except for the medium with 200 mM NaCl where their significant decrease was recorded (Fig. 3). On the other hand, increased levels of bioactive CK forms were found in roots grown on 50, 100 and 150 mM NaCl. Transport, storage and deactivation CKs represent the three elevated groups of CKs in *AtCKX2*-17 shoots grown on all NaCl concentrations. Unlike shoots, the transport, storage and deactivation CK forms exhibited varying dependencies on applied NaCl concentrations in *AtCKX2*-17 roots. In *AtCKX2*-17 shoots, the immediate biosynthetic CK precursors decreased or remained unaffected on NaCl-treated media (except for their increase on 150 mM). On the other hand, the contents of CK phosphates were elevated in roots. In analogy to *AtCKX1*-29 transgenic line, *AtCKX2*-17 shoots and roots showed increased total CKs on all applied NaCl concentrations (with the exception of 200 mM NaCl for roots) in comparison to the control plants grown on NaCl-free medium.

Interestingly, another analysed *AtCKX2* line, *AtCKX2-*26, showed different pattern compared with *AtCKX2*-17 (Fig. 4). Whereas the levels of bioactive CKs were increased on 50 and 100 mM NaCl in transgenic *AtCKX2-*26 shoots, their decrease was recorded on 150 and 200 mM NaCl. Varying dependencies on applied NaCl concentrations were demonstrated for transport, storage, deactivation CK forms as well as for CK phosphates in transgenic *AtCKX2*-26 shoots. In contrast to other centaury lines including both non-transformed and *AtCKX* transformed plants the *AtCKX2*-26 shoots were unique exhibiting decreased total CKs contents on NaCl containing media. In *AtCKX2*-26 roots, the contents of all particular CK forms as well as total CKs were increased on media containing more than 50 mM NaCl.

### Quantification of endogenous IAA levels and determination of IAA/bioactive cytokinins ratio

**Figure 5:**
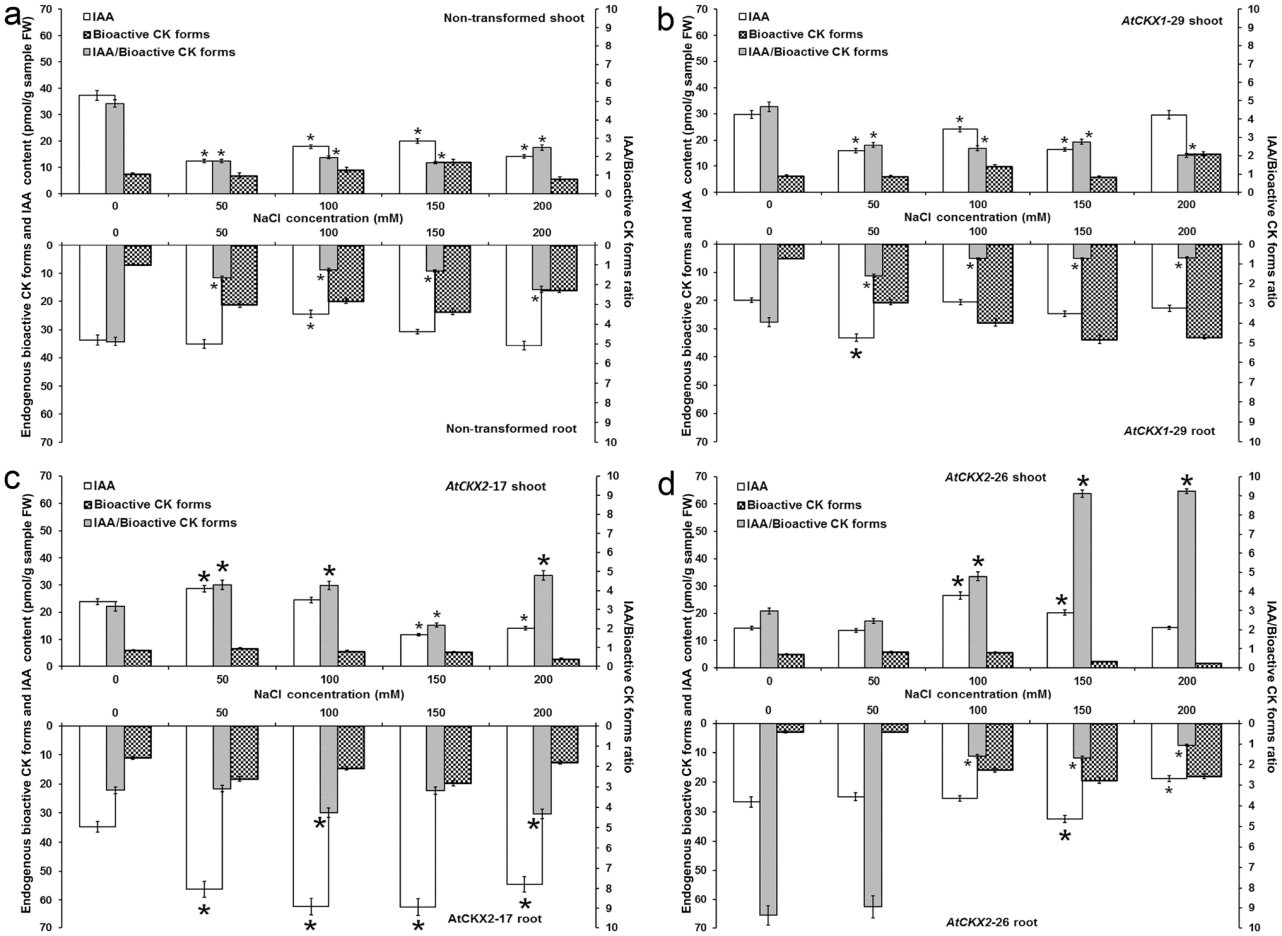
Endogenous IAA and bioactive cytokinin contents and their ratios in shoots and roots of 8-week-old non-transformed (**a**), *AtCKX1*-29 (**b**), *AtCKX2*-17 (**c**) and *AtCKX2*-26 (**d**) transgenic *Centaurium erythraea* lines. Data represent mean ± standard error. Means marked with asterisks are significantly different from corresponding control values (LSD test, *p* ≤ 0.05). The bigger asterisks represent the values significantly higher than control ones while smaller asterisks represent values significantly lower than control ones.

Alterations in endogenous IAA contents were detected in non-transformed and *AtCKX*-overexpressing centaury plants grown in vitro on media with graded NaCl doses (Fig. 5). In non-transformed shoots, the IAA levels were decreased on all applied NaCl concentrations (Fig. 5a). Simultaneously, the contents of bioactive CKs were reduced on 50 and 200 mM NaCl and increased on 150 mM NaCl (Fig. 1). On the other hand, in non-transformed roots a decrease of IAA content was detected only on 100 mM NaCl while the amounts of bioactive CK contents were enhanced on all NaCl concentrations. Regardless these differences, the IAA/bioactive CKs ratios were decreased in all non-transformed shoots as well as in roots on all applied NaCl concentrations (Fig. 5a).

A similar pattern of the IAA contents was ascertained in *AtCKX1*-29 plants (Fig. 5b). In comparison to the NaCl-free medium, *AtCKX1*-29 shoots showed decrease in the IAA contents on 50, 100 and 150 mM NaCl. The levels of bioactive CK forms were elevated on two NaCl concentrations, 100 and 200 mM NaCl (Fig. 2). Unlike shoots, in the *AtCKX1*-29 roots increased IAA contents were detected only on 50 mM NaCl whereas elevation of bioactive CK amounts were recorded on all NaCl concentrations. In analogy to the non-transformed plants, the *AtCKX1*-29 shoots and roots showed decreased IAA/bioactive CKs ratios (Fig. 5b).

In *AtCKX2*-17 shoots, a significant reduction of endogenous IAA contents was recorded on 150 and 200 mM NaCl while an enhancement was found on 50 mM (Fig. 5c). At the same time, the bioactive CK levels were decreased only on 200 mM NaCl (Fig. 3). Consequently, the IAA/bioactive CKs ratios in transgenic *AtCKX2*-17 shoots increased on 50, 100 and 200 mM NaCl while decreased on 150 mM NaCl. In the roots of *AtCKX2*-17, both IAA and bioactive CK detected on all NaCl concentrations (except for bioactive CKs on 200 mM). Accordingly, IAA/bioactive CKs ratios were elevated on 100 and 200 mM NaCl (Fig. 5c).

Endogenous IAA and bioactive CKs contents, as well as IAA/bioactive CKs ratios were considerably altered in transgenic *AtCKX2*-26 plants grown on graded NaCl concentrations (Fig. 5d). In comparison to NaCl-free medium, the *AtCKX2*-26 shoots were characterized by increased IAA content on 100 and 150 mM NaCl. Bioactive CK levels were increased on 50 and 100 mM NaCl and decreased on 150 and 200 mM NaCl concentrations (Fig. 4). Consequently, the IAA/bioactive CKs ratios were almost duplicated on 100 mM and up to triplicated on 150 and 200 mM NaCl compared to the control. In *AtCKX2*-26 roots, the IAA contents were increased on 150 and decreased on 200 mM NaCl whereas the levels of endogenous bioactive CKs were enhanced on all NaCl concentrations exceeding 50 mM. Unlike shoots, the IAA/bioactive CKs ratios in the *AtCKX2*-26 roots were declined in comparison to the control about 5-times on 100 and 150 mM and even 9-times 200 mM NaCl.

### Analysis of endogenous stress hormones

**Figure 6:**
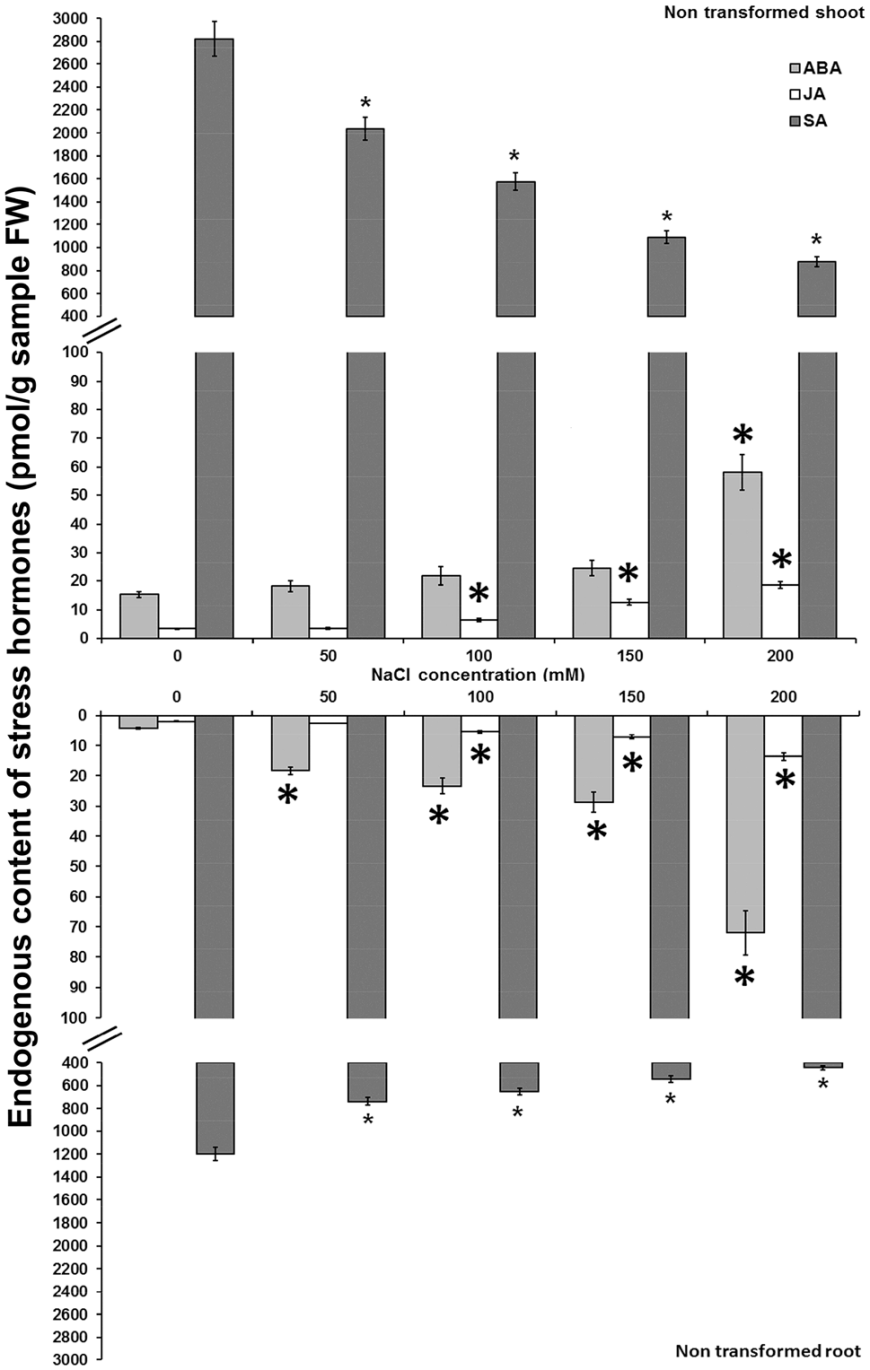
Endogenous ABA, JA and SA contents and in shoots and roots of 8-week-old non-transformed *Centaurium erythraea* line. Data represent mean ± standard error. Means marked with asterisks are significantly different from corresponding control values (LSD test, *p* ≤ 0.05). The bigger asterisks represent the values significantly higher than control ones while smaller asterisks represent values significantly lower than control ones.

**Figure 7:**
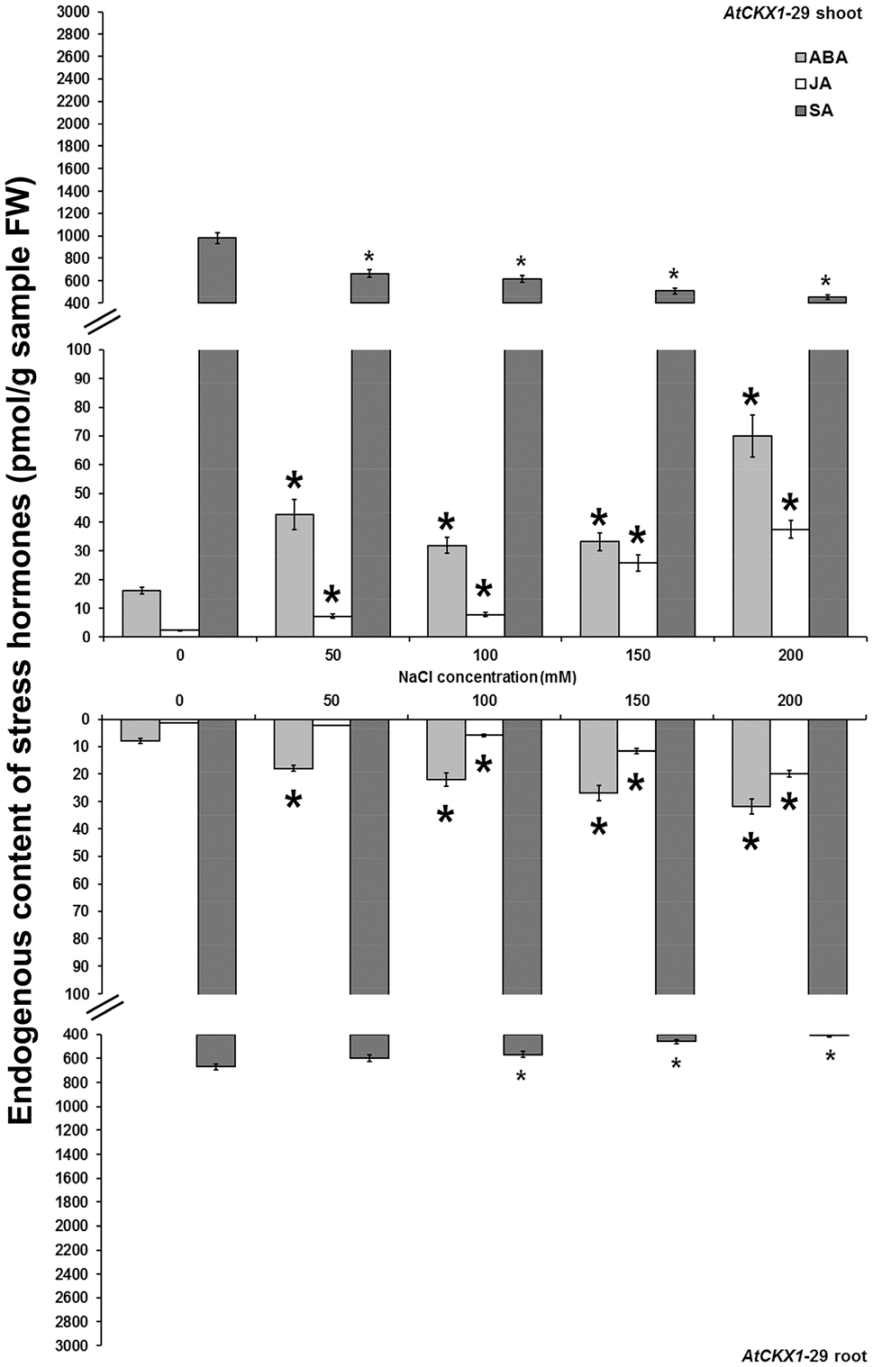
Endogenous ABA, JA and SA contents and in shoots and roots of 8-week-old *AtCKX1*-29 transgenic *Centaurium erythraea* line. Data represent mean ± standard error. Means marked with asterisks are significantly different from corresponding control values (LSD test, *p* ≤ 0.05). The bigger asterisks represent the values significantly higher than control ones while smaller asterisks represent values significantly lower than control ones.

**Figure 8:**
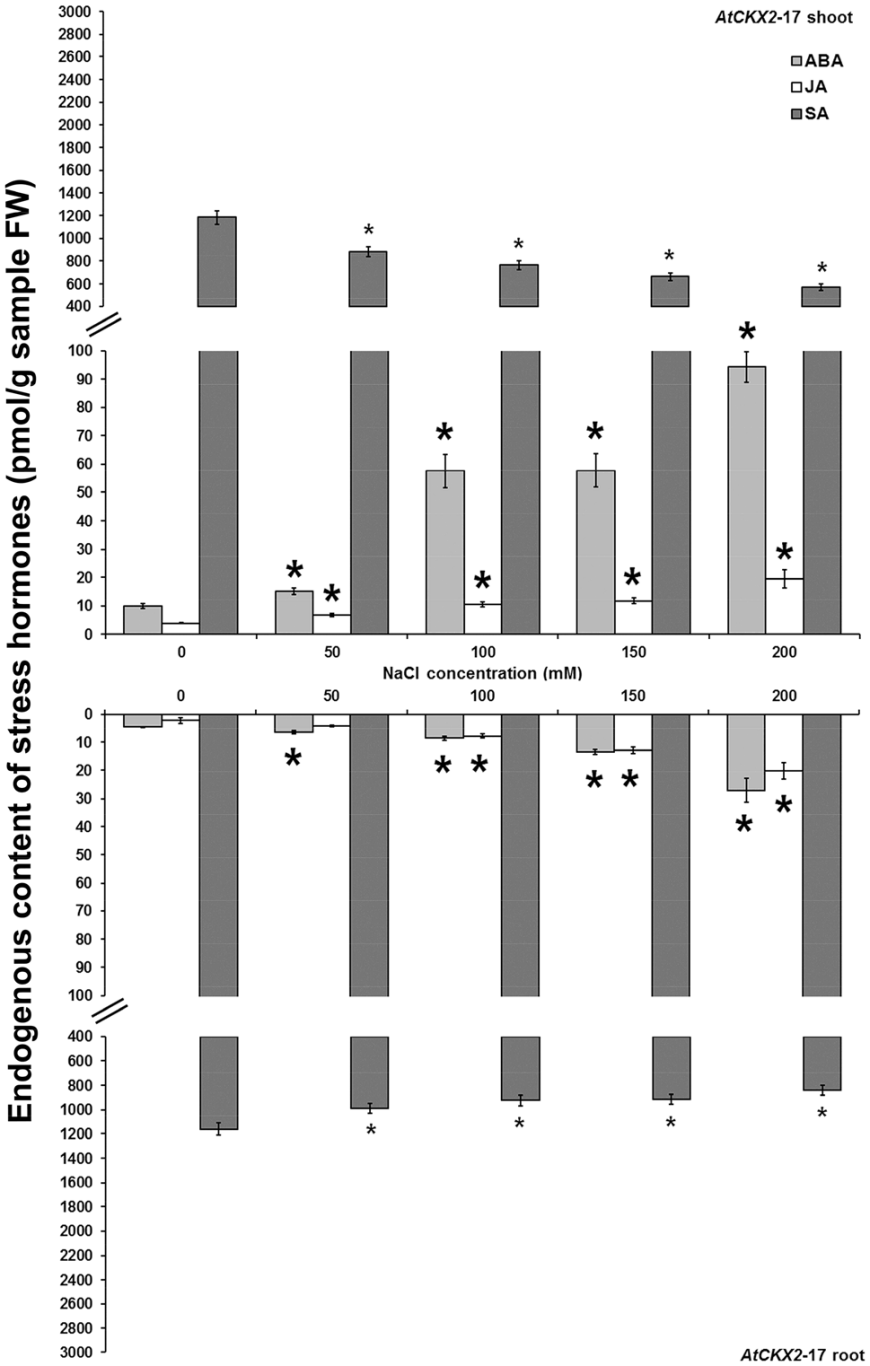
Endogenous ABA, JA and SA contents and in shoots and roots of 8-week-old *AtCKX2*-17 transgenic *Centaurium erythraea* line. Data represent mean ± standard error. Means marked with asterisks are significantly different from corresponding control values (LSD test, *p* ≤ 0.05). The bigger asterisks represent the values significantly higher than control ones while smaller asterisks represent values significantly lower than control ones.

**Figure 9:**
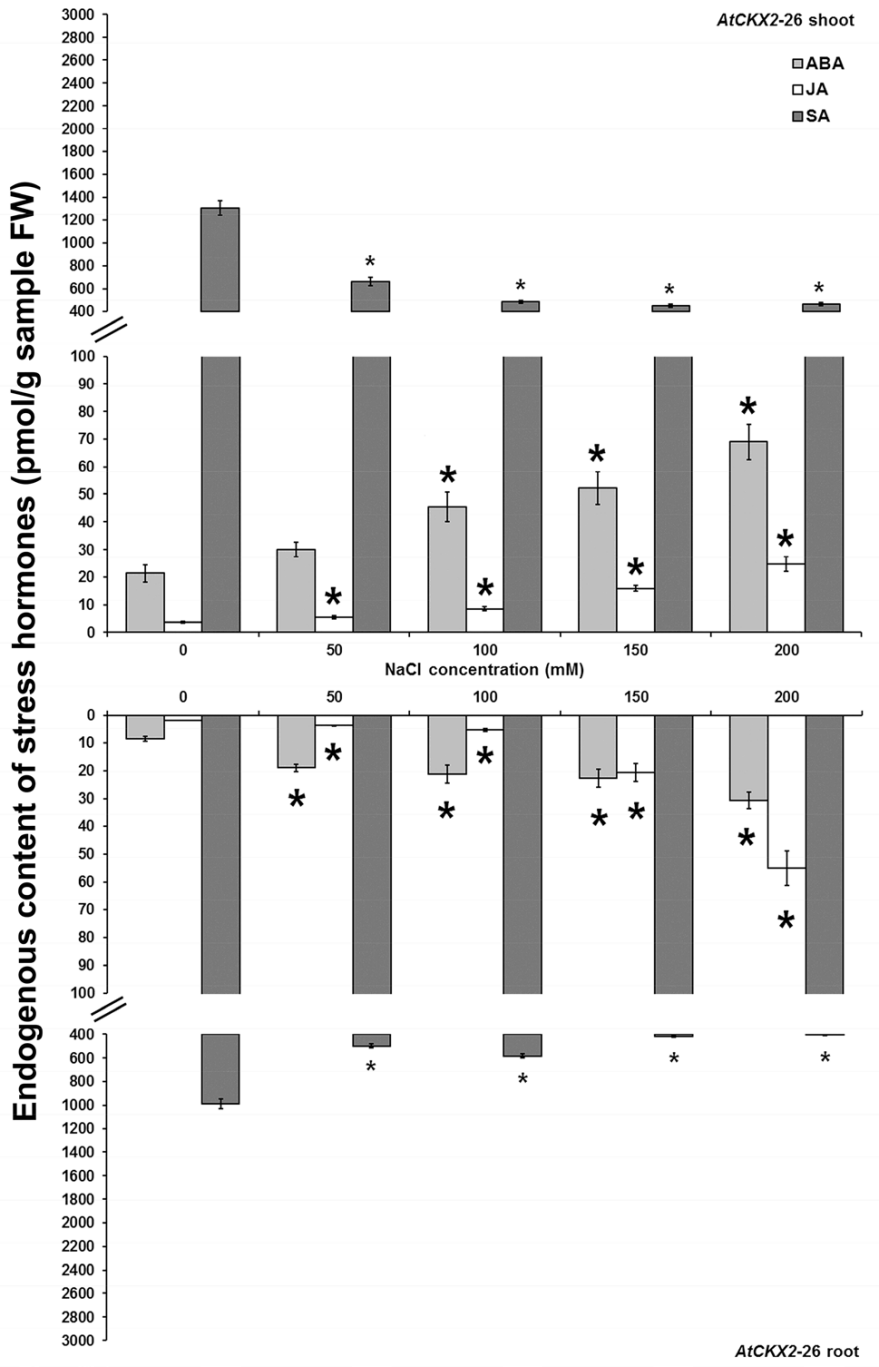
Endogenous ABA, JA and SA contents and in shoots and roots of 8-week-old *AtCKX2*-26 transgenic *Centaurium erythraea* line. Data represent mean ± standard error. Means marked with asterisks are significantly different from corresponding control values (LSD test, *p* ≤ 0.05). The bigger asterisks represent the values significantly higher than control ones while smaller asterisks represent values significantly lower than control ones.

The levels of endogenous stress hormones, ABA, JA and SA, in non-transformed and *AtCKX* transgenic centaury shoots and roots cultured on media with graded NaCl concentrations were also determined (Figs. 6, 7, 8, 9). In all analysed shoot and root samples, the same increment/decrement tendency for particular stress hormone was noticed. The ABA and JA contents were elevated on increased NaCl concentrations in both non-transformed and *AtCKX* transgenic plants reaching the highest levels on 200 mM NaCl. Oppositely, the endogenous SA contents in both control and *AtCKX* plants were decreased on enhanced NaCl concentrations with the lowest level of SA on 200 mM NaCl.

It was intersting to note that in non-transformed shoots JA and SA contents were higher than in roots on all applied NaCl concentrations while ABA content was higher in non-transformed roots than in shoots only on 200 mM NaCl (Fig. 6). On the other hand, transgenic *AtCKX1*-29, *AtCKX2*-17 and *AtCKX2*-26 shoots showed higher ABA and JA contents than roots (Figs. 7, 8, 9). Also, higher SA content were determined in *AtCKX1*-29 shoots than in roots whereas in both *AtCKX2* lines the similar amounts of SA were detected in both organs.

## Discussion

Phytohormones are produced in very low concentrations in plants where they act as chemical messengers in signalling mechanisms under stress conditions. In this study, non-transformed and *AtCKX* transgenic centaury plants were grown on graded NaCl concentrations in vitro with the aim to elucidate how salinity stress affects phytohormone profiles of this valuable medicinal plant species. Taking advantage of advanced high-performance liquid chromatography tandem mass spectrometry methodology, the patterns and levels of endogenous CKs as well as the amounts of auxin IAA, ABA, JA and SA in the shoots and roots of *AtCKX1* and *AtCKX2* centaury lines under salt stress were analyzed and are shown in the present work.

Cytokinins are present in all plant tissues regulating numerous developmental processes such as cell division, apical dominance, leaf senescence, vascular and shoot differentiation and anthocyanin production^3^. It is known that CKs play both positive and negative roles in the stress tolerance. According to various reports, endogenous CK levels were decreased in the course of enhanced stressed conditions^13,42^ while some observations indicated increased CKs content, whether it was short- or long-term exposure to stress^43,44^. The observed changes in the endogenous CK contents clearly indicated an involvement of these hormones in plant stress responses. Plant resistance to stress conditions is a complex process and the role of phytohormones can be investigated e.g. on plants with altered endogenous CK levels. Considering that CKs have significant roles in plant stress responses, genes encoding two key enzymes in CK metabolism, *ipt* (responsible for CK biosynthesis) and *ckx* (responsible for CK degradation), were proposed as possible target genes involved in stress resistance^13,45^. Altered endogenous CK levels in *Arabidopsis*, *Nicotiana tabacum* and *Hordeum vulgare* plants, modified either by loss of the *ipt* genes expression or by overexpression of the *ckx* encoding genes, showed that CKs played a negative role in response to heat and/or drought stress^10,13,45–49^. Also, there are literature data describing that reduced CK contents improved drought and salt stress tolerance in *Arabidopsis ckx* overexpressing lines and *ipt1*, *ipt3*, *ipt5* and *ipt7* mutants^13,45,50^.

Previous investigation showed that overexpression of the *AtCKX1* and *AtCKX2* genes in transgenic centaury plants resulted in altered CKs profile leading to a decline of bioactive CK levels and, at the same time, increased contents of storage CK forms, inactive CK forms and/or CK nucleotides^51^. Previous investigation showed that overexpression of *CKX* genes caused hyposensitivity to salt stress and drought stress in arabidopsis^13^ and *Physcomitrella patens*^52^. Our current results generally indicated that all groups of endogenous CKs were considerably altered in non-transformed and *AtCKX* centaury shoots on all applied NaCl concentrations and that the total CK contents were elevated comparing to the corresponding control. Furthermore, increased levels of all five CK groups as well as of total CKs were recorded on used NaCl concentrations in non-transformed and *AtCKX* centaury roots. Furthermore, analyses of endogenous CKs showed that the levels of bioactive CK forms varied considerably among non-transformed and all analysed *AtCKX* shoots depending on NaCl concentration. On the other hand, increased levels of bioactive CK forms were found in roots of all analysed centaury lines, on almost all applied NaCl concentration. These observations indicated that salinity triggered different changes in the CK contents in centaury shoots and roots. Considering that CKs are produced mainly in roots which are directly involved in responses to nutrients it can be concluded that CKs represent an important signal traveling from roots to the shoots. The transport of CK from roots to shoots is considered as the key factor in response to salt stress^53^. Therefore, the salt-induced changes in CK profiles may disturb homeostasis on the subcellular level and also further alter the CK content in other compartments. Furthermore, it is important to note that altered CK levels in centaury tissues may also be the result of their prolonged exposure to salinity during eight weeks. Any strong conclusions about CK content is very difficult because the complexity of the cytokinin profiles has not been fully explored^53^.

It is known that CKs affect antioxidant systems and related processes under drought and salt stress. Recent biochemical analyses showed lower free proline, MDA content and H_2_O_2_ production as well as reduced SOD and POX activities in transgenic *AtCKX* centaury plants under prolonged salinity conditions^15^. Similarly, the activity of SOD was considerably decreased in leaves and roots of transgenic *AtCKX2* tobacco plants during the salt stress^24^. Previous investigations showed that ectopic expression of *ckx* genes in barley activated genes involved in biosynthesis of flavonoids^49^ and also in drought tolerance^54^. On the other hand, increased level of antioxidants during the stress could protect plant cells from reactive oxygen species (ROS) accumulation and reduce electrolyte leakage and/or rises in malondialdehyde levels^55,56^. All of these results indicated the effects of CKs in stress tolerance and involvement of antioxidant systems in response to manipulation of CK homeostasis. It was also reported that *AtCKX* transgenic centaury roots showed higher salinity tolerance compared to shoots^15^. These results are in accordance with findings describing that the *ckx* overexpression enhanced root growth, nutrient uptake and drought tolerance^54,57^. Additionally, several literature data indicate that the growth and development of root tissues could be important in CK-regulated responses to water limiting conditions, including the differentiation of vascular tissue^58^ and lignification^48^.

Together with CKs, auxins play a crucial role in regulation of plant growth and development. They are also involved in plant tolerance against various environmental stresses like salinity, however, mechanisms of salt stress regulation by these phytohormones are not yet clear enough. Several studies revealed IAA accumulation under salt treatments in *Arabidopsis*, maize and *Prosopis strombulifera*^18,59,60^. A cross-talk between auxins and CKs has been widely studied and possible antagonistic roles in plant defense responses were revealed. Specific mechanisms that modulate this cross-talk are, however, still unknown^61,62^. Our results showed altered endogenous IAA content in shoots and roots of non-transformed as well as *AtCKX*-overexpressing centaury plants grown on graded NaCl concentrations in vitro. Interestingly, non-transformed and *AtCKX1*-29 shoots and roots showed decreased IAA/bioactive ratio in response to salinity whereas this ratio in both *AtCKX2* transgenic lines varied considerably. A close connection between IAA levels and CK contents has been previously reported in *AtCKX1* and *AtCKX2* centaury transformants^51^ that contained lower amounts of endogenous IAA in shoots as well as in roots. Consequently, the IAA/bioactive CKs ratios showed a significant variation in the shoots and roots of all analysed *AtCKX* transformed centaury plants. Previously it was shown that auxins affect CK degradation by downregulating *ckx* genes^63^. Jones et al.^64^ showed that elevated CK level increased auxin biosynthesis in young, developing root and leaf tissues while decreased endogenous CK levels reduced auxin biosynthesis in seedlings and root meristem tissues of *Arabidopsis*. Literature data also demonstrated reduced IAA contents in *AtCKX1* and *AtCKX2 Arabidopsis* seedlings^10^. On the other hand, overexpression of *AtCKX3* transgenes did not affect endogenous IAA content in tobacco leaves^65^. Varying endogenous IAA and CKs contents and ratios may be explained by the fact that auxin-cytokinin cross-talk pathways are dependent on the developmental stage and tissue specific.

Considering that the plant responses to stress conditions involve numerous physiological and biochemical changes, including alterations in concentrations and ratios of endogenous stress phytohormones, it was observed that increasing salinity in centaury plants is associated with an increment of ABA and JA as well as a decrement of SA contents. In non-transformed and transgenic *AtCKX* centaury shoots and roots, ABA and JA levels were elevated on increased NaCl concentrations, reaching the maxima on 200 mM NaCl. ABA is well known to represent an endogenous signal molecule that helps plants to survive unfavorable environmental conditions. Accordingly, the exposure of plants to drought and high salt stress resulted in a proportional enhancement of ABA and JA levels in salt-sensitive and salt-tolerant plant species^66^. Increased endogenous ABA and JA contents were also observed in *Iris hexagona* as a response to salinity^37^. All these reports indicated that higher ABA and JA levels could act as effective protectors against salt stress conditions. Also, there are literature data describing exogenous ABA application in raising the plant salt tolerance^67–69^. Likewise, exogenous application of JA was successfully applied in order to recover the salt-induced defects on plants^6,70,71^. Exogenously amended stress hormones during salt treatment may change the balance of endogenous hormones, and thus provide an important trait for understanding the protection mechanisms against the salt stress. All of these results clearly demonstrate that ABA and JA have positive roles not only during wounding and biotic stress but also as effective protection agents against high salinity.

SA is generally associated with plant biotic stress. However, numerous reports regarding SA participation in plant responses to abiotic stresses, salinity especially, have been documented recently. Our results showed enhanced SA content in non-transformed shoots and roots in comparison to centaury *AtCKX* lines. Some plant species contain very high basal levels of SA and consequently, SA does not appear to act as an effective signal molecule for defense genes and inducing the disease resistance^72^. It is known that CKs enhance the SA response which results in increased transcription of defense-related genes such as *SID2* for salicylic acid biosynthesis and *PR1*, a marker gene for salicylic acid responses^73^. Accoedingly, it was expected that *AtCKX* centaury plants with altered endogenous CK content show different SA content comparing to non-transformed. Our results also showed declined SA contents in shoots and roots of non-transformed as well as transgenic *AtCKX* centaury plants grown on increased NaCl concentrations in vitro. The lowest endogenous SA content was observed on 200 mM NaCl. These results correlate with a reported decline of endogenous SA levels in *Iris hexagona*^37^ and rice seedlings^36^ under salt stress. However, some studies showed that SA accumulation is a signal of injury of plant tissues under adverse environmental conditions^74,75^. Just because some studies have reported the improved stress tolerance by SA accumulation whereas others have demonstrated less stress tolerance when the endogenous SA level remain high, the role of SA in plant responses to abiotic stress is not clear yet. It is also reported that SA considerable cross-talk with other plant hormones is implicated in drought and salinity tolerance mechanisms. In general, antagonistic interactions between SA and ABA are well known^76,77^. Beside, numerous literature data are describing that exogenous application of SA elevated salt stress resistance. The ameliorative effects of SA have been well documented including salt tolerance in crops such as bean^78^ and mustard^79^. All of these described studies demonstrated that endogenous SA level has a plastic response that is modified depending on the plant organ, particular species or exposed type of stress. Considering that plants are continuously subjected to exposure to multiple stresses under natural conditions, endogenous hormones need to be interconnected in order to allow them to coordinate the reactions leading to survival using often limited resources. Exploring the interaction of endogenous phytohormones still remains a quite issue considering that all phytohormones excessively involved in cross-talk with each other and also affect and alter each other biosynthesis.

## Conclusion

Altered contents and ratio of endogenous phytohormones such as CKs, auxin IAA, ABA, JA and SA in non-transformed and *AtCKX* transgenic lines indicated their involvement in responses of centaury plants to salinity. Considering that *AtCKX* transgenic centaury plants grow on medium supplemented with graded NaCl concentrations but without exogenous application of plant growth regulators, it can be concluded that endogenous phytohormones act as a main determinant for the salt tolerance. The majority of plant hormonal investigations are based on their exogenous applications but the knowledge of changing endogenous phytohormone profiles in response to salt stress during plant growth still remains limited. Although it is known that different plant hormones cross-talk mutually, the mechanisms underlying in these complex signal pathways have not yet been described thoroughly. Unravelling the mechanisms of different phytohormone actions undoubtedly represents a promising tool for future investigations.

## Methods

### Plant material and culture conditions

All experiments were performed with plant material originating from *C. erythraea* Rafn. seeds obtained from Jelitto Staudensamen GmbH, Schwarmstedt, Germany. Transgenic *AtCKX* centaury (*Centaurium erythraea* Rafn.) plants were obtained as previously described^80^. Shoots and roots of non-transformed, one transgenic *AtCKX1* (*AtCKX1*-29) and two *AtCKX2* (*AtCKX2*-17 and *AtCKX2*-26) centaury lines (≈ 10 mm long) were separately cultured in vitro, on half-strength MS medium (½MS^81^). Shoot explants (10) were cultured in 1 l bottles with 100 ml of culture media, while root explants (20–25) were grown in petri dishes (∅ 10 cm) filled with 25 ml of culture media. The selection of three transgenic lines was based on overexpression of *AtCKX* genes in centaury shoots and roots which resulted in an altered CKs profile leading to a decline of bioactive CK levels and, at the same time, increased contents of storage CK forms, inactive CK forms and/or CK nucleotides^51^. The medium was solidified with 0.7% agar and supplemented with 3% sucrose and 100 mg l^−1^ *myo*-inositol. All explants were cultured on ½MS hormone free or ½MS medium supplemented with graded NaCl concentrations (50, 100, 150 and 200 mM). The medium was adjusted to pH 5.8 with NaOH/HCl and autoclaved at 121 °C for 25 min. All in vitro cultured plants were grown at of 25 ± 2 °C and a 16 h/8 h photoperiod (“Tesla” white fluorescent lamps, 65 W, 4500 K; light flux of 47 μmols^−1^ m^−2^). **A**ll methods were performed in accordance with the relevant guidelines and regulations.

### Extraction, purification and quantification of endogenous phytohormones

Endogenous CKs, auxins, ABA, JA and SA were analysed in extracts of 8-week-old in vitro-grown shoot and root explants of non-transformed and *AtCKX* transgenic centaury lines. The plant explants were extracted, purified and quantified according to Dobrev and Kamínek^82^ and Dobrev et al.^83^. Briefly, samples (≈100 mg FW) were homogenised with 1.3 mm silica beads using a FastPrep-24 instrument (MP Biomedicals, CA, USA) with extraction buffer methanol/H_2_O/formic acid (15:4:1, v:v:v) supplemented with stable isotope-labeled internal standards, each at 10 pmol per sample. The extracts were subjected to solid phase extraction (SPE) using Oasis-MCX cartridges (Waters, Milford, MA, USA), with two resulting fractions: (1) fraction A (eluted with methanol) containing hormones of acidic and neutral character (IAA, ABA, JA and SA); and (2) fraction B (eluted with 0.35 M NH4OH in 70% methanol) containing hormones of basic character (CKs). The SPE eluates were evaporated to dryness and pellets reconstituted in 30 μl of 5% methanol in water. An aliquot (10 µL) of the extract was injected on an Ultimate 3000 high-performance liquid chromatograph (Dionex, Bannockburn, IL, U.S.A.) coupled to a 3200 Q TRAP hybrid triple quadrupole/linear ion trap mass spectrometer (Applied Biosystems, Foster City, CA, U.S.A.) set in selected reaction monitoring mode. Quantitative analysis was done using isotope dilution method with multilevel calibration curves (r2 > 0.99). Data processing was carried out with Analyst 1.5 software (Applied Biosystems). The concentration of all analysed phytohormones are presented as pmol/g of sample fresh weight.

### Statistical analysis

The effect of graded NaCl concentrations (0–200 mM) on endogenous phytohormones content of 8-week-old non-transformed and transgenic *AtCKX* shoots and roots were evaluated using standard two-factor analysis of variance (ANOVA). Determination of endogenous phytohormones in three biological samples (for shoots and roots) per non-transformed and each transgenic line was repeated twice. Results are presented as mean ± SE. The comparisons between the mean values were made using a Fisher LSD (the least significant difference) post-hoc test calculated at a confidence level of *p* ≤ 0.05.

## Abbreviations

*c*Z: *cis*-zeatin
*c*Z7G: *cis*-zeatin 7-glucoside
*c*Z9G: *cis*-zeatin 9-glucoside
*c*ZOG: *cis*-zeatin *O*-glucoside
*c*Z9R: *cis*-zeatin 9-riboside
*c*ZRMP: *cis*-zeatin 9-riboside-5′-monophosphate
*c*Z9ROG: *cis*-zeatin 9-riboside *O*-glucoside
DHZ: Dihydrozeatin
DHZ7G: Dihydrozeatin 7-glucoside
DHZ9G: Dihydrozeatin 9-glucoside
DHZOG: Dihydrozeatin *O*-glucoside
DHZ9R: Dihydrozeatin 9-riboside
DHZRMP: Dihydrozeatin 9-riboside-5′-monophosphate
DHZ9ROG: Dihydrozeatin 9-riboside *O*-glucoside
iP: *N*^*6*^-(∆^2^-isopentenyl)adenine
iP7G: *N*^*6*^-(∆^2^-isopentenyl)adenine 7-glucoside
iP9G: *N*^*6*^-(∆^2^-isopentenyl)adenine 9-glucoside
iP9R: *N*^*6*^-(∆^2^-isopentenyl)adenine 9-riboside
iPRMP: *N*^*6*^-(∆^2^-isopentenyl)adenine 9-riboside-5′-monophosphate
*t*Z: *trans*-zeatin
*t*Z7G: *trans*-zeatin 7-glucoside
*t*Z9G: *trans*-zeatin 9-glucoside
*t*ZOG: *trans*-zeatin *O*-glucoside
*t*Z9R: *trans*-zeatin 9-riboside
*t*ZRMP: *trans*-zeatin 9-riboside-5′-monophosphate
*t*Z9ROG: *trans*-zeatin 9-riboside *O*-glucoside

## References

[1] Hussain M (2017). Rice in saline soils: Physiology, biochemistry, genetics, and management. Adv. Agron..

[2] Nguyen D, Rieu I, Mariani C, van Dam NM (2016). How plants handle multiple stresses: Hormonal interactions underlying responses to abiotic stress and insect herbivory. Plant. Mol. Biol..

[3] Fahad S (2015). Phytohormones and plant responses to salinity stress: A review. Plant Growth Regul..

[4] Bielach A, Hrtyan M, Tognetti VB (2017). Plants under stress: Involvement of auxin and cytokinin. Int. J. Mol. Sci..

[5] Mok DW, Mok MC (2001). Cytokinin metabolism and action. Annual Rev. Plant Physiol. Plant Mol. Biol..

[6] Javid MG, Sorooshzadeh A, Moradi F, Sanavy SAMM, Allahdadi I (2011). The role of phytohormones in alleviating salt stress in crop plants. Aust. J. Crop Sci..

[7] Galuszka P (2007). Biochemical characterization of cytokinin oxidases/dehydrogenases from *Arabidopsis thaliana* expressed in *Nicotiana tabacum* L. Plant Growth Regul..

[8] Gajdošová S (2011). Distribution, biological activities, metabolism, and the conceivable function of *cis*-zeatin-type cytokinins in plants. J. Exp. Bot..

[9] Schmülling T, Werner T, Riefler M, Krupková E, Bartrina y Manns I (2003). Structure and funcyion of cytokinin oxidase dehydrogenase genes of maize, rice, *Arabidopsis* and other species. J. Plant Res..

[10] Werner T (2003). Cytokinin-deficient transgenic Arabidopsis plants show multiple developmental alterations indicating opposite functions of cytokinins in the regulation of shoot and root meristem activity. Plant Cell.

[11] Tran LS, Shinozaki K, Yamaguchi-Shinozaki K (2010). Role of cytokinin responsive two-component system in ABA and osmotic stress signalings. Plant Signal Behav..

[12] Nishiyama R (2012). Transcriptome analyses of a salt-tolerant cytokinin-deficient mutant reveal differential regulation of salt stress response by cytokinin deficiency. PLoS ONE.

[13] Nishiyama R (2011). Analysis of cytokinin mutants and regulation of cytokinin metabolic genes reveals important regulatory roles of cytokinins in drought, salt and abscisic acid responses, and abscisic acid biosynthesis. Plant Cell.

[14] Žižková E (2015). Tomato (*Solanum lycopersicum* L.) SlIPT3 and SlIPT4 isopentenyltransferases mediate salt stress response in tomato. BMC Plant Biol..

[15] Trifunović-Momčilov M (2020). Salinity stress response of non-transformed and AtCKX transgenic centaury (*Centaurium erythraea* Rafn.) shoots and roots grown in vitro. Ann. App. Biol..

[16] Lau S, Jurgens G, De Smet I (2008). The evolving complexity of the auxin pathway. Plant Cell.

[17] Petersson SV (2009). An auxin gradient and maximum in the *Arabidopsis* root apex shown by high-resolution cell-specific analysis of IAA distribution and synthesis. Plant Cell.

[18] Wang Y, Li K, Li X (2009). Auxin redistribution modulates plastic development of root system architecture under salt stress in *Arabidopsis thaliana*. J. Plant Physiol..

[19] Kazan K (2013). Auxin and the integration of environmental signals into plant root development. Ann. Bot..

[20] Gharbi E (2017). Phytohormone profiling in relation to osmotic adjustment in NaCl-treated plants of the halophyte tomato wild relative species *Solanum chilense* comparatively to the cultivated glycophyte *Solanum lycopersicum*. Plant Sci..

[21] Vishwakarma K (2017). Abscisic acid signaling and abiotic stress tolerance in plants: A review on current knowledge and future prospects. Front. Plant Sci..

[22] Cutler SR, Rodriguez PL, Finkelstein RR, Abrams SR (2010). Abscisic acid: Emergence of a core signaling network. Annu. Rev. Plant Biol..

[23] Kim TH, Böhmer M, Hu H, Nishimura N, Schroeder JI (2010). Guard cell signal transduction network: Advances in understanding abscisic acid, CO2, and Ca2+ signaling. Annu. Rev. Plant Biol..

[24] Mýtinová Z (2010). Effect of abiotic stresses on the activity of antioxidative enzymes and contents of phytohormones in wild type and *AtCKX2* transgenic tobacco plants. Biol. Plant..

[25] Pospíšilová J (2003). Interaction of cytokinins and abscisic acid during regulation of stomatal opening in bean leaves. Photosynthetica.

[26] Wang Y (2011). Cytokinin antagonizes ABA suppression to seed germination of Arabidopsis by downregulating ABI5 expression. Plant J..

[27] Verslues PE (2016). ABA and cytokinins: Challenge and opportunity for plant stress research. Plant Mol. Biol..

[28] Jeon J (2010). A subset of cytokinin two-component signaling system plays a role in cold temperature stress response in *Arabidopsis*. J. Biol. Chem..

[29] Kumar MN, Verslues PE (2015). Stress physiology functions of the *Arabidopsis* histidine kinase cytokinin receptors. Physiol. Plantarum.

[30] Brugière N (2003). Cytokinin oxidase gene expression in maize is localized to the vasculature, and is induced by cytokinins, abscisic acid, and abiotic stress. Plant Physiol..

[31] Nazar R, Iqbal N, Syeed S, Khan NA (2011). Salicylic acid alleviates decreases in photosynthesis under salt stress by enhancing nitrogen and sulfur assimilation and antioxidant metabolism differentially in two mungbean cultivars. J. Plant Physiol..

[32] Saraf, R., Saingar, S., Chaudhary, S. & Chakraborty, D. Response of plants to salinity stress and the role of salicylic acid in modulating tolerance mechanisms: Physiological and proteomic approach in Biotic and abiotic stress tolerance in plants (ed Vats, S.) 103–136 (Springer, 2018).

[33] Rajeshwari V, Bhuvaneshwar V (2017). Enhancing salinity tolerance in Brinjal plants by application of salicylic acid. J. Plant Sci..

[34] Mohammadi M, Chaichi MR, Safikhani S (2019). Salicylic acid application alleviates the salt stress effects in wheat. Int. J. Dev. Res..

[35] Shaki F, Maboud HE, Niknam V (2019). Effects of salicylic acid on hormonal cross talk, fatty acids profile, and ions homeostasis from salt-stressed safflower. J. Plant Inter..

[36] Sawada H, Shim IS, Usui K (2006). Induction of benzoic acid 2-hydroxylase and salicylic acid biosynthesis modulation by salt stress in rice seedlings. Plant Sci..

[37] Wasternack C, Hause B (2002). Jasmonates and octadecanoids: Signals in plant stress responses and plant development. Prog. Nucleic Acid Res. Mol. Biol..

[38] Wang Y, Mopper S, Hasentein KH (2001). Effects of salinity on endogenous ABA, IAA, JA, and SA in *Iris hexagona*. J. Chem. Ecol..

[39] Pedranzani H (2003). Salt tolerant tomato plants show increased levels of jasmonic acid. Plant Growth Regul..

[40] Lomin SN (2015). Plant membrane assays with cytokinin receptors underpin the unique role of free cytokinin bases as biologically active ligands. J. Exp. Bot..

[41] Kamínek M (2000). Purine cytokinins: A proposal for abbreviations. Plant Growth Regul..

[42] Merewitz EB, Gianfagna T, Huang B (2011). Photosynthesis, water use, and root viability under water stress as affected by expression of SAG12-ipt controlling cytokinin synthesis in *Agrostis stolonifera*. J. Exp. Bot..

[43] Alvarez S, Marsh EL, Schroeder SG, Schachtman DP (2008). Metabolomic and proteomic changes in the xylem sap of maize under drought. Plant Cell Environ..

[44] Dobra J (2010). Comparison of hormonal responses to heat, drought and combined stress in tobacco plants with elevated proline content. J. Plant Physiol..

[45] Werner T (2010). Root-specific reduction of cytokinin causes enhanced root growth, drought tolerance, and leaf mineral enrichment in Arabidopsis and Tobacco. Plant Cell.

[46] Macková H (2013). Enhanced drought and heat stress tolerance of tobacco plants with ectopically enhanced cytokinin oxidase/dehydrogenase gene expression. J. Exp. Bot..

[47] Lubovská Z, Dobrá J, Štorchová H, Wilhelmová N, Vanková R (2014). Cytokinin oxidase/dehydrogenase overexpression modifies antioxidant defense against heat, drought and their combination in *Nicotiana tabacum* plants. J. Plant Physiol..

[48] Pospíšilová H (2016). Transgenic barley overexpressing a cytokinin dehydrogenase gene shows greater tolerance to drought stress. New Biotechnol..

[49] Vojta P (2016). Whole transcriptome analysis of transgenic barley with altered cytokinin homeostasis and increased tolerance to drought stress. New Biotechnol..

[50] Li S (2019). Overexpression of the cytokinin oxidase/dehydrogenase (CKX) from *Medicago sativa* enhanced salt stress tolerance of Arabidopsis. J. Plant Biol..

[51] Trifunović M (2015). Changes in cytokinin content and altered cytokinin homeostasis in AtCKX1 and AtCKX2-overexpressing centaury (*Centaurium erythraea* Rafn.) plants grown in vitro. Plant Cell Tiss. Organ Cult..

[52] Hyoung S (2020). Cytokinin oxidase PpCKX1 plays reg-ulatory roles in development and enhances dehydration and salt tolerance in *Physcomitrella patens*. Plant Cell Rep..

[53] Schachtman DP, Goodger JQ (2008). Chemical root to shoot signaling under drought. Trends Plant Sci..

[54] Nakabayashi R (2014). Enhancement of oxidative and drought tolerance in Arabidopsis by overaccumulation of antioxidant flavonoids. Plant J..

[55] Liao X (2017). Overexpression of MsDREB6.2 results in cytokinin-deficient developmental phenotypes and enhances drought tolerance in transgenic apple plants. Plant J..

[56] Ramireddy E (2018). Root engineering in barley: Increasing cytokinin degradation produces a larger root system, mineral enrichment in the shoot and improved drought tolerance. Plant Physiol..

[57] Xu S (2017). Wild tobacco genomes reveal the evolution of nicotine biosynthesis. Proc. Natl. Acad. Sci. USA.

[58] Jang G, Choi YD (2018). Drought stress promotes xylem differentiation by modulating the interaction between cytokinin and jasmonic acid. Plant Signal Behav..

[59] Zörb C, Geilfus CM, Mühling K, Ludwing-Müller J (2013). The influence of salt stress on ABA and auxin concentrations in two maize cultivars differing in salt resistance. J. Plant Physiol..

[60] Llanes A, Masciarelli O, Ordoñez R, Isla MI, Luna V (2014). Differential growth responses to sodium salts involve different ABA catabolism and transport in the halophyte *Prosopis strombulifera*. Biol. Plantarum.

[61] Bishopp A, Benková E, Helariutta Y (2011). Sending mixed messages: Auxin-cytokin in crosstalk in roots. Curr. Opin. Plant. Biol..

[62] Vanstraelen M, Benková E (2012). Hormonal interactions in the regulation of plant development. Annu. Rev. Cell Dev. Biol..

[63] Werner T, Köllmer I, Bartrina I, Holst K, Schmülling T (2006). New insights into the biology of cytokinin degradation. Plant Biol..

[64] Jones B (2010). Cytokinin regulation of auxin synthesis in Arabidopsis involves a homeostatic feedback loop regulated via auxin and cytokinin signal transduction. Plant Cell.

[65] Polanská L (2007). Altered cytokinin metabolism affects cytokinin, auxin, and abscisic acid contents in leaves and chloroplasts, and chloroplast ultrastructure in transgenic tobacco. J. Exp. Bot..

[66] Prerostova S (2017). Hormonal dynamics during salt stress responses of salt-sensitive *Arabidopsis thaliana* and salt-tolerant *Thellungiella salsuginea*. Plant Sci..

[67] Yang Z, Yu L, Merewitz E, Huang B (2012). Differential effects of abscisic acid and glycine betaine on physiological responses to drought and salinity stress for two perennial grass species. J. Am. Soc. Hortic. Sci..

[68] Sripinyowanicha S (2013). Exogenous ABA induces salt tolerance in indica rice (*Oryza sativa* L.): The role of OsP5CS1 and OsP5CR gene expression during salt stress. Environ. Exp. Bot..

[69] Sales L (2018). Salt tolerance in apple seedlings is affected by exogenous ABA application. Acta Hortic..

[70] Qiun ZB, Guo JL, Zhu AJ, Zhang L, Zhang MM (2014). Exogenous jasmonic acid can enhance tolerance of wheat seedlings to salt stress. Ecotoxicol. Environ. Saf..

[71] Yuan F, Liang X, Li Y, Yin S, Wang B (2018). Methyl jasmonate improves tolerance to high salt stress in the recretohalophyte *Limonium bicolor*. Fun. Plant. Biol..

[72] Yang Y, Qi M, Mei C (2004). Endogenous salicylic acid protects rice plants from oxidative damage caused by aging as well as biotic and abiotic stress. Plant J..

[73] Zubo YO, Schaller GE (2020). Role of the cytokinin-activated type-B response regulators in hormone crosstalk. Plants.

[74] Hao L (2011). Role of endogenous salicylic acid in *Arabidopsis* response to elevated sulfur dioxide concentration. Biol. Plant..

[75] Devinar G, Llanes A, Masciarelli O, Luna V (2013). Abscisic acid and salicylic acid levels induced by different relative humidity and salinity conditions in the halophyte *Prosopis strombulifera*. Plant Growth Regul..

[76] Miura K, Sato A, Ohta M, Furukawa J (2011). Increased tolerance to salt stress in the 873 phosphate-accumulating Arabidopsis mutants siz1 and pho2. Planta.

[77] La VH (2019). Antagonistic shifting from abscisic acid- to salicylic acid-mediated sucrose accumulation contributes to drought tolerance in *Brassica napus*. Environ. Exp. Bot..

[78] Azooz MM (2009). Salt stress mitigation by seed priming with salicylic acid in two faba bean genotypes differing in salt tolerance. Int. J. Agric. Biol..

[79] Syeed S (2011). Salicylic acid-mediated changes in photosynthesis, nutrients content and antioxidant metabolism in two mustard (*Brassica juncea* L.) cultivars differing in salt tolerance. Acta Physiol. Plant..

[80] Trifunović M (2013). Overexpression of *Arabidopsis* cytokinin oxidase/dehydrogenase genes *AtCKX1* and *AtCKX2* in transgenic *Centaurium erythraea* Rafn. Plant Cell Tiss. Organ Cult..

[81] Murashige T, Skoog F (1962). A revised medium for rapid growth and bioassays with tobacco tissue cultures. Physiol. Plantarum.

[82] Dobrev PI, Kamínek M (2002). Fast and efficient separation of cytokinins from auxin and abscisic acid and their purification using mixed-mode solid phase extraction. J. Chromatogr. A.

[83] Dobrev, P.I., Hoyerova, K., Petrasek, J. Analytical Determination of Auxins and Cytokinins in *Auxins and Cytokinins in Plant Biology: Methods and Protocols, Book Series: Methods in Molecular Biology* vol. 1569, (eds. Dandekar, T., Naseem, M.) 31–39 (Springer, 2017). 10.1007/978-1-4939-6831-2_2 28265985

